# Internet usage among the oldest-old: does functional health moderate the relationship between internet usage and autonomy?

**DOI:** 10.1007/s10433-023-00748-z

**Published:** 2023-02-21

**Authors:** Veronica Oswald, Michael Wagner

**Affiliations:** 1 Cologne Center for Ethics, Rights, Economics, and Social Sciences of Health, University of Cologne, Universitätsstraße 91, 50931 Cologne, Germany; 2 Faculty of Management, Economics and Social Sciences, Institute of Sociology and Social Psychology (ISS), University of Cologne, Cologne, Germany

**Keywords:** Information and communication technology (ICT), Active aging, Compensation, Subjective well-being

## Abstract

In recent years, research on internet usage in old age and its associations with well-being outcomes has increased. However, the oldest-old age group (80 years and older) is frequently underrepresented, and autonomy and functional health are rarely considered in these studies. By applying moderation analyses with a representative dataset of the oldest-old in Germany (*N* = 1863), our study has analyzed the hypothesis that the autonomy of older individuals, in particular of those with limited functional health, can be enhanced by internet usage. The moderation analyses indicate that the positive association between internet usage and autonomy is greater for older individuals with lower functional health. This association remained significant after controlling for social support, housing situation, education, gender, and age. Explanations for these results are discussed, and imply that further research is needed to understand the relationships between internet usage, functional health, and autonomy.

## Introduction

Maintaining autonomy is a key issue in active aging that is of crucial interest for both individuals and policymakers. In gerontology, “autonomy is the perceived ability to control, cope with and make personal decisions about how one lives on a day-to-day basis, according to one’s own rules and preferences” (WHO 2017). Maintaining autonomy improves outcomes like well-being and life satisfaction (cf. Hüning et al. 2018; Niemiec and Ryan 2009). The usage of information and communications technologies (ICT) in old age can help individuals maintain their autonomy, and, in turn, their quality of life (cf. Cotton et al. 2014; Szabo et al.^2019^). ICT usage (and technology use in general) by older individuals has considerable development potential in the domains of physiological and psychological health, everyday activities and leisure, mobility, social involvement, and security (Schmidt and Wahl 2019). Web-based ICT usage offers older individuals new forms of social participation and interaction, and enhances their access to information, services, and entertainment (Antonucci et al. 2017; Caruso 2018).

Many studies have investigated the relationship between internet usage and well-being outcomes, and have found positive effects (cf. Cotten et al. 2014; Szabo et al. 2019). However, compared with other indicators of well-being, the autonomy of older individuals has been rarely investigated. Moreover, in the studies that have considered autonomy, it was analyzed as one of several well-being indicators, and not as a separate factor (e.g. Szabo et al. 2019). A few studies have found a relationship between internet usage and higher levels of autonomy among older individuals (e.g. Hartanto et al. 2020; Schlomann et al. 2020). Other studies have shown that older individuals who use the internet see it as a resource for maintaining autonomy in old age, and for dealing with aging-related and general challenges, such as banking or understanding English (Nimrod 2020; Seifert and Schelling 2018). However, earlier studies found no evidence for an effect of internet usage on older individuals’ autonomy (e.g. Slegers et al. 2008).

Research on internet usage in old age has mainly focused on its well-being outcomes, without taking older individuals’ health into account (cf. Forsman et al. 2018), even though there is a high prevalence of functional health limitations in older age groups (Marengoni et al. 2011; Wolff et al. 2017). Maintaining autonomy with restricted health is challenging (Baltes and Silverberg 1994; Schüz et al. 2011). In particular, having low levels of functional health restricts the scope of individual’s daily activities. To maintain an autonomous life, individuals have to cope with the challenges of limited health (Baltes and Silverberg 1994; Schüz et al. 2011). For example, individuals who are restricted in walking may be unable to do grocery shopping without help. The internet is a potential resource for helping such individuals cope with this constraint. One option is to order the groceries for delivery via the internet. Older individuals may have a stronger sense of autonomy if they are able to take action and make their own grocery choices. This is just one example of a range of opportunities for exercising autonomy via the internet.

A review of the current state of research on maintaining autonomy despite limited health shows that studies on this topic are often intervention studies that examine special health applications (eHealth; WHO 2017). The evidence for effects reported in eHealth studies is training or support effects, rather than effects of the internet usage itself (Dickinson and Gregor 2006). A study that took functional limitations into account without a focus on eHealth found that older individuals in residential care facilities who use the internet reported higher levels of autonomy and life satisfaction than those who do not use the internet (Seifert et al. 2017). The authors concluded that the internet is a resource, especially for older individuals, for coping with the challenges of vulnerability. These findings are in line with the results of Fang et al. (2018), who investigated internet usage by older individuals and their psychological well-being. According to the authors, the evidence for a positive effect of internet usage only applies to individuals aged 75 years or older, and especially to frail individuals. It has been suggested that internet usage can help to compensate for the functional limitations of the very old (Wangler and Jansky 2020).

In sum, the current state of research shows that the relationship between internet usage and autonomy in old age has hardly been investigated in detail, especially for older individuals with health limitations and the often underrepresented group of the oldest-old. The few studies that have investigated the well-being outcomes of internet usage by the oldest-old lacked representativeness and statistical tests, or left unclear whether the ICT usage was web-based (e.g. Sims et al. 2017). It is reasonable to assume that internet usage becomes more important to individuals in old age, as it helps them to compensate for the decline in the capabilities that they require to perform everyday activities, and to interact with the (social) environment in accordance with their own needs (cf. Wahl et al. 2010). Against this background, we aim to analyze the relationship between internet usage, autonomy, and functional health in the oldest-old age group. Our research question is whether internet usage by the oldest-old is related to their perceived autonomy, and whether this relationship is moderated by their functional health.

Human Development Theory assumes that human development is incomplete, and that developmental gains and losses vary within an individual’s life span (Baltes 1997). First, it is assumed that with increasing age, the number of dysfunctional gene expressions increases. As a result of these dysfunctional gene expressions, the prevalence of diseases such as dementia increases with old age. Additionally, the need for psychological, social, material, and knowledge-based resources grows. To maintain high levels of functioning in old age, individuals need these resources to compensate for their developmental losses (e.g., health-related losses). Lastly, the theory assumes that there is a reduction in the efficiency of the aforementioned resources across an individual’s life span (e.g., a reduction in the effectiveness of cognitive learning). To summarize, Human Development Theory asserts that with increasing age, individuals will experience more developmental losses than gains.

Accordingly, the internet as a (environmental) resource can contribute to an individual’s ability to cope with age-related functional limitations while maintaining his or her autonomy. Baltes (1997) observed that in addition to the mechanisms of optimization and selection, compensation is often used to regulate functional losses in old age. The related strategy for aging successfully is to select a limited number of activities, and to optimize them to compensate for age-related losses. For example, older individuals could select the internet for online banking or online shopping in order to save time and energy (optimization), as well as to compensate for their restricted mobility (cf. Nimrod 2020).

The theoretical assumptions of Human Development Theory allow us to hypothesize that the assumed positive association between internet usage and autonomy is moderated by functional health. The internet can be categorized as an environmental resource, while limited functional health can be considered a state of reduced personal resources. Human Development Theory assumes that when individuals’ personal resources, such as their (functional) health, are declining, they have a greater need for environmental resources to cope with the challenges they face in maintaining their autonomy. Thus, the internet is an external resource that can help individuals to maintain their autonomy via selection, optimization, and compensation processes. Furthermore, because the profound challenges individuals with functional health limitations face mean that they are more dependent on the support of the environment to maintain their autonomy, the utility of the internet should be higher for individuals with functional limitations than for individuals without these limitations. The association between internet usage and autonomy should become stronger with lower levels of functional health. Accordingly, our hypotheses are as follows: H1: Internet usage by the oldest-old is positively associated with their autonomy. H2: The association between internet usage and autonomy becomes greater with lower functional health.

## Method and research design

### Data and study sample

Data from a representative survey of adults aged 80 years and older (born before August 1, 1937) in North Rhine–Westphalia, a federal state of Germany, were employed. The survey on “NRW80+” (Wagner et al. 2018) was conducted from August 2017 to February 2018 (see Zank et al. 2020). The study was funded by the Ministry of Culture and Science of the German State of North Rhine–Westphalia. The computer-assisted face-to-face interviews were conducted in the homes of the target persons, and covered a variety of topics, ranging from family, health, living conditions, social relations, individual values, and well-being to daily activities and lifestyle. The dataset includes 1878 individuals who were living in private homes, as well as individuals who were living in institutional care settings. The multistage sampling was conducted in two steps. First, a sample of 93 communes was drawn from all communes in North Rhine–Westphalia. Second, a random sample of 48137 addresses was provided by the population registration offices, of which 8040 have been contacted. Apart from the age and the principal residence, there were no additional exclusion criteria. A total of 1702 interviews were conducted with the target individuals, and 176 interviews were conducted with a proxy informant to include individuals who were not able to answer the questions because of health impairments.

## Measures

### Dependent variable

Perceived autonomy was measured with a single item based on the autonomy scale by Schwarzer (2008). The corresponding question was: “Do you lead your life according to your own ideas?” The response scale was a four-point Likert scale, ranging from “does not apply at all” to “fully applies.” As the planned duration of the interviews was 90 min, autonomy was included as a single item instead of as multiple items, based on the economic method in survey research. Because the items within the scale by Schwarzer (2008) show high internal consistence (*α* = 0.90), the single item should sufficiently represent the multiple-item scale.

### Independent variables

Internet usage was measured by asking whether the participant had used the internet in the last 12 months. The possible answers were “yes” or “no.” The variable internet usage was dummy coded.

For measuring functional health, the “activities of daily living” (ADL) scale was included (McDowell 2006).^1^ The scale measures seven items of activities of daily living: eating, dressing, body care, walking, bathing, and toilet use. Participants were asked whether they can perform the respective activity “only with help,” “with little help,” or “without help.” A mean score was built from the seven items ranging from zero to two, with higher values indicating higher functional health.

### Control variables

Research has shown that social support can promote autonomy (e.g. Warner et al. 2011). Furthermore, studies have found that social support can positively affect the usage of ICT, including of the internet (Hänninen et al. 2021). Thus, received social support was included in the analysis. Participants were asked how often in the past 12 months they had received social support from other persons in the form of “being comforted or cheered up” and “getting help with tasks and errands (excluding paid help).” The possible answers ranged from “never” to “always” on a five-point scale. Based on the two items, a mean score ranging from zero to two was computed.

Individuals’ living environment may also influence their internet usage in old age (e.g., Seifert et al. 2017). Moreover, individual’s living environment can affect their autonomy. Compared to living in a long-term care institution, living in a private household tends to offer people greater autonomy, as it provides them with more scope for taking action and making self-determined decisions (Hajek et al. 2021; Schüz et al. 2013). Thus, the living environment was included in the analysis by using the housing situation measure, which indicated whether the participant was living in a long-term care institution or in a private home. The variable was dummy coded.

Individuals’s demographic characteristics, such as their age, gender, and education, may also influence their functional health and internet usage. Age seems to negatively influence internet usage; men use the internet more often than women; and higher education seems to influence internet usage positively (cf. Hunsaker and Hargittai 2018; König et al. 2018). In addition, functional limitations seem to be more prevalent in women than in men; age positively influences the occurrence of functional limitations; and higher education seems to buffer the occurrence of functional limitations due to higher awareness of health behaviors (Marengoni et al. 2011; Wolff et al. 2017). Therefore, age, gender, and education were considered in the current analysis. Age was measured continuously in years. Gender was measured with a categorical variable (“male”[ref.] or “female”), and the level of education was measured based on the International Standard Classification of Education (ISCED) (“low”[ref.], “middle,” or “high”). For the moderation analysis, the education variable was recoded into three dummy variables.

## Statistical analyses

SPSS version 27 (IBM Statistics) and the PROCESS macro version 3.5.3 were used for the statistical analyses. Missing values were excluded listwise. All analyses included 1772 cases with valid values for all variables.^2^ First, descriptive analyses were applied. Subsequently, the moderation model was initially conducted without covariates. In a second step, the control variables were included in the moderation model. To analyze whether the association between internet usage and autonomy becomes greater with lower functional health, model 1 of the PROCESS macro was used^3^ with bootstrapping 10000 times and 95% confidence intervals. In terms of traceability, the seed was set to 10821. Based on the Breusch–Pagan test, heteroscedasticity could not be rejected (model without covariates: *χ*^2^(1) = 242.85, *p* < 0.001; model with covariates: *χ*^2^(1) = 167.91, *p* < 0.001). Thus, heteroscedasticity consistent standard errors were used in the moderation models. To probe the interaction effect, the mean value, the value of one standard deviation below the mean, and the value of one standard deviation above the mean were used. Additionally, to test whether significance transition points exist within the observed range of the moderator functional health, the Johnson–Neyman method was used.

## Results

### Descriptive statistics

The mean age in the sample was 86.97 years (SD = 4.50). The participants’ ages ranged from 80 to 103 years. In terms of gender, 50.73% of the sample were female and 49.27% were male. In terms of education, 26.69% had low education, 52.09% medium education, and 21.22% high education. While 10.21% of the participants were living in a long-term care institution, the majority (89.79%) were living in a private home. The mean value of autonomy (ranging from one to four) was relatively high, at 3.43 (SD = 0.85). Only 20.15% of the participants reported using the internet, while 79.85% indicated that they were not using the internet. The mean value of functional health was 1.63 (SD = 0.53) (ranging from zero to two). The mean value of social support was 0.57 (SD = 0.25) (ranging from zero to two).

Table 1 shows the statistically significant different mean values and distributions in percent when comparing internet users and non-users based on individual and environment-related characteristics. These bivariate analyses indicated that compared to non-users, internet users had statistically significant higher levels of autonomy and functional health, were receiving less social support, were more likely to be living in a private home, had higher education, and were more likely to be male and to be younger.

**Table 1:** Internet usage by sample characteristics

Internet usage by sample characteristics		Internet usage	No internet usage	*t*/*χ*^2^
Total	%	20.15	79.85	–
Autonomy	M(SD)	3.80 (.49)	3.34 (.89)	− 9.33 (1770), *p* < .001
Functional health	M(SD)	1.88 (.30)	1.57 (.56)	− 10.09 (1770), *p* < .001
Social support	M(SD)	.53 (.28)	.58 (.24)	3.30 (1770), *p* < .001
Age	M(SD)	85.12 (3.83)	87.44 (4.54)	8.89 (1770), *p* < .001
Education	Low (%)	4.23	95.77	241.21 (2), *p* < .001
	Medium (%)	17.55	82.45	
	High (%)	46.54	53.46	
Gender	Male (%)	30.24	69.76	108.98 (1), *p* < .001
	Female (%)	10.34	89.66	
Housing	Private home (%)	22.19	77.81	40.32 (1), *p* < .001
	Long-term care institution (%)	2.21	97.79	

*N* = 1772. 2-tailed significance with 95% confidence intervals. *χ*^2^-tests were applied for the categorical variables gender, education, and housing. *T* tests were applied for autonomy, functional health, social support, and age. *M* mean, *SD* standard deviation

Moreover, the bivariate correlations showed a statistically significant positive correlation between internet usage and autonomy (*r* = 0.22, *p* < 0.001), and between functional health and autonomy (*r* = 0.57, *p* < 0.001) (Table 4). The positive correlation between functional health and internet usage (*r* = 0.23, *p* < 0.001) indicated that the internet was more likely to be used as a resource by individuals with higher levels of functional health. Referring to the control variables, housing was relatively highly correlated with autonomy (*r* = − 0.45, *p* < 0.001) and functional health (*r* = − 0.43, *p* < 0.001). Likewise, education was relatively highly correlated with internet usage (*r* = 0.36, *p* < 0.001). However, to test the hypothesis that the association between internet usage and autonomy became greater with functional limitations, further multivariate investigations were conducted.

## Moderation models

The aim of the applied moderation models was to examine the extent to which the differences in autonomy levels between internet users and non-internet users were a function of their functional health. Table 2 displays the two moderation models. While the first model did not include the covariates, the second model included the covariates. The final model explained 38.06% of the variance in autonomy. The interaction of internet usage and functional health, as well as all of the other conditional associations, was statistically significant. This means that the positive association between internet usage and autonomy was stronger at lower levels of functional health, and was weaker at higher levels of functional health. The interaction coefficient estimated the difference in autonomy levels between internet users and non-users as the value of functional health increased by the value of one. Accordingly, with an increase in the value of one on the functional health scale, the difference in the association between internet usage and non-internet usage decreased by 0.33 scale points of autonomy.

**Table 2:** Functional health on the relationship between internet usage and autonomy

Independent variables	*M*1			*M*2			
	*b* (SE)	*t*	*p*	*b* (SE)	*t*	*p*	*B*
Constant	1.93 (.08)	24.43	< .001	2.15 (.36)	6.03	< .001	.02
${b}_{1}:$ Internet usage (ref. no usage)	1.07 (.31)	3.45	< .001	.77 (.30)	2.52	< .05	.11
${b}_{2}:\mathrm{ Functional health}$	.90 (.04)	21.13	< .001	.72 (.05)	15.66	< .001	.42
${b}_{3}:$ Internet usage x Functional health	− .47 (.16)	− 2.96	< .01	− .33 (.16)	− 2.10	< .05	− .08
*Conditional effects of internet usage at values of functional health*							
${b}_{m-SD}:$ mean-SD	.55 (.14)	4.02	< .001	.41 (.13)	3.02	< .01	.19
${b}_{m}:$ mean	.30 (.06)	5.20	< .001	.23 (.06)	3.93	< .001	.11
${b}_{m+SD}:$ mean + SD	.12 (.03)	3.72	< .001	.11 (.04)	3.05	< .01	.05
*Control variables*							
Social support	–	–	–	− .10 (.06)	− 1.69	.091	− .03
Housing (ref. private home)	–	–	–	− .68 (.07)	− 9.17	< .001	− .25
Education level: medium (ref. low)	–	–	–	.02 (.04)	.51	.607	.01
Education level: high (ref. low)	–	–	–	.04 (.05)	.64	.520	.02
Gender (ref. male)	–	–	–	.03 (.04)	.71	.479	.02
Age	–	–	–	.00 (.00)	.44	.662	.01
Model fit	*N* = 1772			*N* = 1772			
	*F*(3, 1768) = 197.05, *p* < .001, *R*^2^ = .33			*F*(9, 1762) = 86.75, *p* < .001, *R*^2^ = .38			
	Change in *R*^2^: *F*(1, 1768) = 8.78, *p* < .01, *R*^2^ = .01			Change in *R*^2^: *F*(1, 1762) = 4.40, *p* < .05, *R*^2^ = .00			

Heteroscedasticity consistent standard errors (HC3), 95% confidence intervals, and 10,000 bootstrap samples are used. *b* unstandardized regression coefficient. *SE* standard error. *ref*. reference. *B* standardized coefficients

This decreasing association was probed with the conditional association of internet usage at certain values of functional health. Therefore, the mean value of functional health and the values one standard deviation below and above the mean value of functional health were used. The model predicted that under the condition that someone had the mean level of functional health, internet usage increased his or her autonomy by 0.23 scale points compared to non-internet usage. If the value of functional health was one standard deviation below the mean, this association was greater ($b_{{m\, - \,{\text{SD}}}}$ = 0.41, *p* < 0.01), and if the value of functional health was one standard deviation above the mean, the association was smaller ($b_{{m\, + \,{\text{SD}}}}$ = 0.11, *p* < 0.01). These estimated conditional associations indicated that the association between internet usage and autonomy became greater with lower levels of functional health.

It is important to note that the coefficients of internet usage und functional health did not represent main effects, but were instead conditional effects. Thus, the coefficient of internet usage indicated that under the condition that individuals had low levels of functional health (the value of functional health was zero), those who were internet users were predicted to have a 0.77 higher value than non-internet users on the autonomy scale. The coefficient of functional health indicated that for non-internet users (the value of internet usage was zero), autonomy was predicted to increase by 0.72 scale points if functional health increased by the value of one.

Based on the Johnson–Neyman method, the observed range of the moderator (values from zero to two on the functional health scale) did not entail significance transition points. Thus, the moderation was statistically significant for all observed values of functional health. In sum, the described moderation models provided evidence of a stronger association between internet usage and autonomy when functional health was low. None of the control variables were significantly associated with autonomy, except the housing situation (*b* = − 0.68, *p* < 0.001). Individuals who were living in a long-term care institution had a significantly lower level of autonomy than individuals who were living in a private home.

**Fig. 1:**
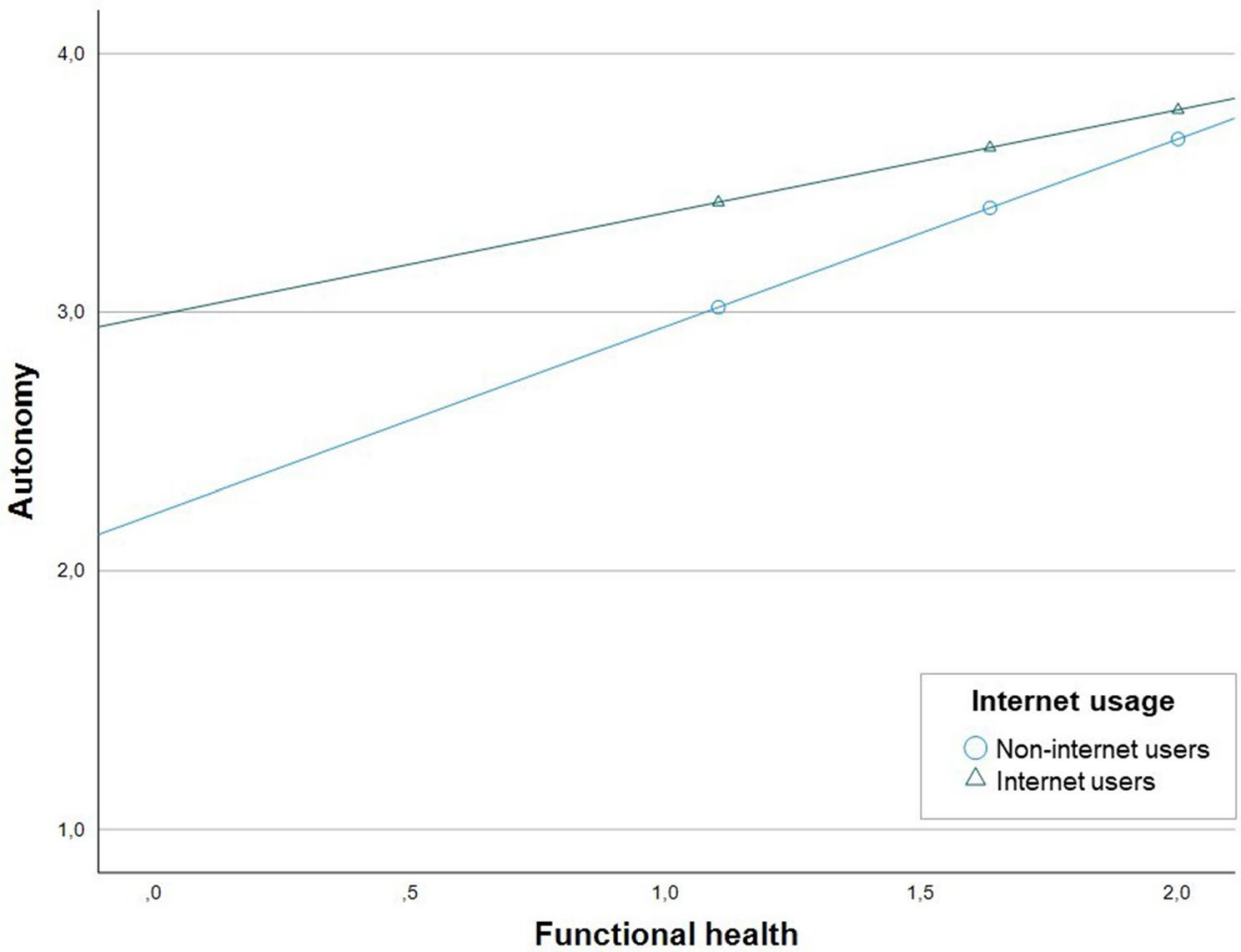
Moderation of functional health on the relationship between internet usage and autonomy

Figure 1 illustrates the conditional associations of the final moderation model. The graphs reflect the conditional associations between functional health and autonomy. The graph with triangles represents the association of functional health with autonomy under the condition that an individual was using the internet, and the graph with circles represents the association of functional health with autonomy under the condition that an individual was not using the internet. The triangles and circles represent the mean value, one standard deviation below and above the mean value of functional health. The positive association between internet usage and autonomy is reflected by the gap between the two graphs. With increased functional health, the gap, and thus the association between internet usage and autonomy, became smaller. Accordingly, the estimated association between internet usage and autonomy was stronger for individuals with a lower level of functional health than for individuals with a higher level of functional health.

## Discussion

This paper aimed to answer the question of whether internet usage by the oldest-old is associated with their autonomy, and whether this relationship is moderated by their functional health. Human Development Theory was used to derive the hypotheses about the relationship between internet usage, functional health, and autonomy. Accordingly, we assumed that internet usage predicts a higher level of autonomy than non-usage, and that this association becomes greater with a lower level of functional health. The bivariate analyses indicated that internet users had a significantly higher level of autonomy than non-users. Moreover, the moderation analyses provided evidence of a positive association between internet usage and autonomy, which became greater with lower functional health. These results are in line with those of prior research (Fang et al. 2018; Seifert et al. 2017) and can be interpreted within the presented theoretical framework that individuals with increasingly challenging functional health limitations are more dependent on the physical–social environment. Thus, for these individuals, the internet has greater utility as a resource.

While the internet is certainly not the only resource older individuals use to cope with the challenge of maintaining their autonomy while having limited functional health (cf. Warner et al. 2011), it can contribute to the coping process. This is also indicated by the small increase in the explained variance by the interaction with less than 1% in both models. However, the interaction remained significant after controlling for covariates. The descriptive results showed that only 20% of the participants in the sample were using the internet. This low usage rate is in line with the findings of previous research on the “grey digital divide” (Friemel 2016; Morris 2007). This research found that because the internet is a relatively new medium, older individuals have had fewer opportunities to evaluate this environmental factor. Diffusion Theory assumes that older individuals are the last group to adopt new technologies in a society (Rogers 2003). However, older individuals may start to use a technology if they perceive it to be useful (BMFSFJ 2010).

For the present analyses, we used functional health as a moderator. In the method section, we argued that functional health is a more appropriate measure. However, it could be argued that multimorbidity is more appropriate, because it is associated with more heterogenic challenges for autonomy than simply the decline in functional health (e.g., mental health outcomes, health care use) (cf. Smith et al. 2018). Therefore, we have also calculated the models with multimorbidity as the moderator variable. The moderation was significant in the first model, but became insignificant after controlling for the covariates. Therefore, it can be assumed that the challenge of declining functional health is more likely to be tackled with internet usage than the other challenges of multimorbidity, or that the number of a person’s chronic diseases is not a valid measure for capturing the impact of the diseases. This assumption is underlined by the high correlation of functional health with autonomy (*r* = 0.57, *p* < 0.001), and the relatively low correlation of autonomy with multimorbidity (*r* = − 0.15, *p* < 0.001).

## Strengths and limitations

The present investigation makes an important contribution to the current state of research, because the previous studies have rarely examined the association between internet usage and autonomy in detail, and health limitations have rarely been included in these studies. Additionally, a representative sample of the frequently underrepresented oldest-old age group was examined. Even oldest-old individuals who were not able to answer the questions themselves could be included through the use of proxy interviews. These proxy interviews were included in the present analysis in the interests of representativeness.

Our data contained a number of missing values within the education variable (cf. Table 3). These missing data could lead to biased coefficient estimates and inaccurate hypotheses tests (Newman 2014). To rule out this possibility, we calculated the model without the education variable. The results are similar to those of the main model.

Another limitation refers to the measurement of autonomy by using a single item. This parsimonious usage of an autonomy measurement may have affected the validity of the measure. However, the high internal consistence of the items in the overall scale indicates that the single item provided sufficient representation. In measuring internet usage, the analysis did not address the frequency and the purpose of internet usage. According to prior research, the purpose as well as the frequency can be crucial for measuring the association between internet usage and well-being outcomes (e.g., Erickson and Johnson 2011; Szabo et al. 2019). Likewise, as the current results are based on cross-sectional data, the calculated associations represent patterns, and not causal relationships. It is also conceivable that higher levels of autonomy and/or functional health predict the probability of internet usage. The moderation of functional health helps to shed light on the association between internet usage and autonomy, but it does not contribute to the clarification of the causal direction. Additionally, as was already noted by other researchers, there might be selection effects in the structure of internet users and non-internet users (cf. Schlomann et al. 2020). In our analyses, we tried to control for such effects by including the moderator of functional health and control variables. For example, it is reasonable to assume that there is more scope for older individuals to make self-determined decisions, and therefore to maintain their autonomy, if they are living in a private household rather than in a long-term care institution (cf. Hajek et al. 2021; Schüz et al. 2013). Although the included control variables aimed to control for such effects, there might be additional selection effects. For example, internet self-efficacy is a crucial factor in internet usage, and helps to explain why the internet is rarely used in old age, even though the potential for usage is high (Jokisch et al. 2021).

## Implications and directions for further research

Based on our results and the outlined limitations, further research should investigate the relationship between internet usage and autonomy in the oldest-old age group, with a moderation of functional health using longitudinal data, to improve our understanding of the direction of the relationship between internet usage, autonomy, and functional health. Moreover, the use of experimental designs to rule out confounding variables is proving helpful for clarifying reverse causation (cf. Hartanto et al. 2021).

Some practical implications can be derived from the evidence showing a stronger association between internet usage and autonomy among individuals with lower functional health. Our results indicate that the “grey digital divide” has not yet been overcome in the oldest-old age group, even though the potential benefits of internet usage are especially large for individuals with functional health limitations. Based on the theoretical and empirical reasoning that older individuals use the internet to cope with developmental losses, policies should aim to promote interest in internet usage by highlighting the (age- and health-related) opportunities it provides. One example is a brochure published by a federal ministry that emphasizes the opportunities of internet use, and that provides answers to several questions about the internet (BMFSFJ 2019). Moreover, as other researchers have stated, “[…] the increasing prevalence of virtual health care services and considering at the same time the comparatively large prevalence of multimorbidity and functional limitations […] does strongly speak for the necessity of developing interventions with the purpose of providing internet access that are tailored to this specific group” (Huxhold et al. 2020). Furthermore, to promote internet competencies, learning strategies should be adapted to the preferences of older individuals (e.g., guided learning or self-regulated learning) (cf. Schlomann et al. 2022).

## Conclusion

The current study analyzed the association between internet usage and autonomy among the often underrepresented oldest-old age group by taking their functional health limitations into account. The investigation has provided robust evidence for a moderation by functional health on the association between internet usage and autonomy. Our findings contribute to the small number of studies on the relationships between internet usage, health status, and autonomy in the oldest-old age group, and provides directions for further research. More research is needed to understand the conditions under which internet usage can promote autonomy in old age. Such research is important, because the internet is an extremely diverse medium that is constantly changing. Internet usage can contribute to active aging and autonomy in old age by optimizing opportunities for improving health, participation, and security.

## Appendix

**Table 3:** Missing values of all included variables

	Autonomy	Internet usage	Functional health	Social support	Housing	Gender	Age	Education
Valid	1860	1862	1863	1861	1858	1863	1857	1783
Missings	3	1	0	2	5	0	6	80

**Table 4:** Zero-order correlations between all included variables

	1	2	3	4	5	6	7
1. Autonomy							
2. Functional health	.57**						
3. Internet usage	.22**	.23**					
4. Social support	− .12**	− .16**	− .08**				
5. Housing	− .45**	− .43**	− .15**	.06**			
6. Education	.15**	.20**	.36**	− .09**	− .12**		
7. Gender	− .11**	− .13**	− .25**	.12**	.15**	− .41**	
8. Age	− .20**	− .30**	− .21**	.08**	.25**	− .08**	.07**

*N* = 1772. ** Correlation is significant at the level .001 level (2-tailed) significant

## Footnotes

1 The health measure multimorbidity was also available in the dataset. However, we have decided to use functional health instead of multimorbidity, because the number of diagnosed diseases is often not in line with the perceived complaints and disabilities (Kuhlmey and Tesch-Römer 2013). Due to advancing medical technology, more diseases can be diagnosed, but they do not necessarily affect the functionality of an individual.

2 Besides single missing values in the included variables, education has a total of 80 missing values (cf. Table 3).

3 PROCESS provides several moderation and mediation models. Model 1 calculates a simple moderation model.
